# Developing Domains and Items about Self-Management among Elderly People with Chronic Disease

**DOI:** 10.3390/healthcare10010054

**Published:** 2021-12-29

**Authors:** Gain Shin, Hae Yean Park

**Affiliations:** 1 Department of Research, VHS Education and Research Institute, Korea Veterans Health Service, Suwon 16275, Korea; cohandi2003@gmail.com; 2 Department of Occupational Therapy, College of Software and Digital Healthcare Convergence, Yonsei University, Wonju 26493, Korea

**Keywords:** chronic disease, domain, eldely, self-management, occupational therapy

## Abstract

Lifestyle is considered as a key factor that affects one’s health and quality of life, and it has become the focus of increasing research interest worldwide. Objectives: We aimed to determine the areas of self-management necessary as part of occupational therapy for elderly people suffering from chronic diseases living in local communities, as well as elements to be included in each area. Method: Delphi survey methodology was utilized. Participants answered three surveys, and we derived the mean, standard deviation, and content validity ratios for each domain and item. Results: We derived 13 domains and 68 items about self-management, derived the fit and importance of 1 domain and 23 items, and finally derived 12 domains and 54 items. Conclusion: The program developed using this research can become a systematic and evidence-based intervention and provide an opportunity for self-management to the target population.

## 1. Introduction

The world population is aging, and the number of individuals over 60 is estimated to increase from 841 million in 2013 to more than 2 billion by 2050. This means that one in nine people is an older person aged 60 years or older [1,2]. As life expectancy increases, the number of older adults suffering from chronic diseases increases. According to a survey, the average number of chronic diseases experienced by an elderly person is 2.7, and 51% of the elderly have three or more chronic diseases [3]. Currently, healthcare resources around the world are focused on the coronavirus disease (COVID-19) [4]. This is in part because a significant portion of the population, up to 25% of people in the United Kingdom, for instance, is designated as high-risk, which includes elderly people aged over 70 and those with underlying health conditions such as respiratory or cardiovascular diseases and cancer [5]. These resource rearrangements could disrupt the continuum of care for elderly people with chronic diseases, so the need to focus on preventing chronic diseases is increasing. Chronic diseases call attention to overall health problems in the elderly, including physical, cognitive, and mental diseases. Accordingly, the prevention and management of chronic diseases for the elderly are discussed as an essential social issue [6]. According to studies, 80% of chronic diseases can be delayed or prevented with good health behaviors such as drug management, self-management (e.g., exercise), and lifestyle changes (e.g., developing healthy eating habits) [7].

Self-management is a strategy of psychological behavior that comprehensively accepts an individual’s internal, external, and environmental factors. Previous studies have stated that self-management is a process of self-discipline in various fields that changes one’s behavior using various strategies or techniques to achieve individual goals [8,9,10]. Thus, for elderly people suffering from chronic diseases, effective self-management not only helps in health maintenance but also provides economic benefits by reducing medical expenses [11,12]. Therefore, many self-management programs are being developed by researchers.

According to previous studies in the field of occupational therapy, research on occupation-based healthcare programs for older adults with chronic diseases is increasing. Such programs include a fall prevention program [13], an occupation-based intervention program for the improvement of daily life activities for elderly people [14], and an occupation-based program for elderly people with chronic pain [15]. The necessity and importance of occupation-based healthcare for the elderly are increasing in the occupational therapy field. In addition, based on a survey of older adults in a community conducted to assess the needs of self-management programs, 64 elderly respondents (94.1%) answered that they wanted the program very much, and 91.2% expressed willingness to participate in the program [16]. In sum, the need for an evidence-based self-management program for people with multiple chronic diseases is on the rise from the perspective of occupational therapy.

Grounds for developing a systematic self-management program can be established through preliminary studies. The Delphi survey method is used to verify the validity of the content to be derived from a questionnaire survey and is conducted with a group of experts who are fit for the purpose of the survey [16,17]. This study used a Delphi survey as the preliminary study to develop a systematic program.

Occupational therapy could play an important role in maintaining independent living of elderly people in the community [18]. The goal of this study was to determine what areas of self-management are necessary for occupational therapy of elderly people suffering from chronic diseases and living in local communities, as well as what should be included in each area. Occupational therapists with a specialty in the community and the elderly were selected for an expert panel, and the Delphi survey method was used to find results that can be used as basic data for inclusion in the future development of self-management programs by occupational therapists.

## 2. Materials and Methods

### 2.1. Study Design

We used the Delphi survey method, a methodology that uses a survey to obtain a group consensus among experts through a series of structured open and closed questions [17,19]. The survey was collected from participant by e-mail in Word format and was replied to only by the researcher’s e-mail. The survey was administered only in South Korea.

### 2.2. Participants

We aimed to recruit occupational therapists who were experts on the elderly population. For this, the researchers invited occupational therapists who had more than three years of experience in research, education, or other relevant areas.

The inclusion criteria were that the participants needed to be experts in the relevant field and had to (1) be able to participate in the Delphi survey within a two-month time frame, (2) complete three stages of the Delphi survey, and (3) have access to email.

All participants included filled out the survey questions during a two-month period (20 August to 17 October 2019). The researchers sent an invitation email introducing the survey to participants. Participants replied with their consent. After completion of all the stages of the survey, each participant received a $5 gift card.

### 2.3. Delphi Method Procedures

Once the participants provided their consent, their demographic information was collected. Twelve key domains of self-management were determined based on the literature review and the previous study about the module used in the Lifestyle Redesign study [15]. Participants were shown a document online consisting of the 12 domains.

**Figure 1:**
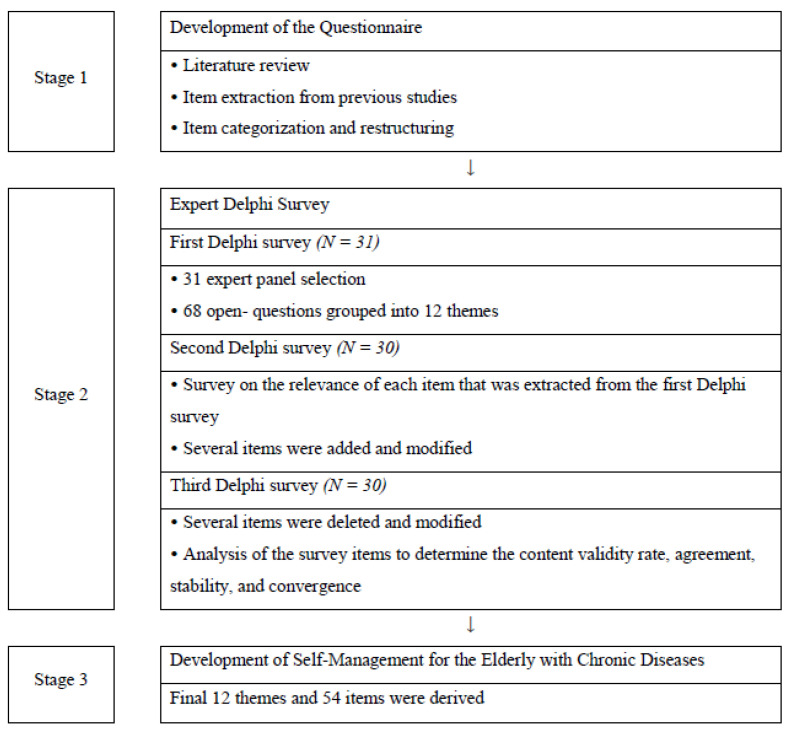
Overall Delphi survey research process consisting of three stages.

The Delphi survey comprised three stages (Figure 1). The participants initiated the first-phase questionnaire by email. The questionnaire consisted of the demographic information of the experts, the need for occupational therapy in each domain, the content to be included in the domain, and questions about occupational therapy activities in the domain. The survey for each phase was available for two weeks. If a deadline passed without a response, a reminder was sent the day after via email and participants were given three additional days to respond.

### 2.4. First Delphi Survey

The first Delphi survey consisted of closed-ended questions grouped into 12 domains and open-ended questions about items to be included in the domains. All open-ended questions were included to ensure that the survey accepted the opinions from the experts. After completing the questionnaire, the participants sent an email to the researchers. Participants were advised to append any recommendations or opinions about the questionnaire. The first stage of the questionnaire required about 20 min to complete.

### 2.5. Second Delphi Survey

The second survey was developed based on the participants’ responses in phase 1. The phase 2 survey consisted of 68 closed-ended questions grouped into 13 domains (physical activity, ADL and IADL management, community integration, medication management, leisure activity management, energy and fatigue management, eating routines, body mechanics and posture, time management, sleep management, social/relationship management, stress and mood management, and paid or unpaid work). The participants received the questionnaire in an email and were required to score the fit and necessity of each domain as well as each proposed element using a five-point Likert-type scale.

### 2.6. Third Delphi Survey

In the third survey, one domain and nine items from the second phase were excluded, and no questionnaire item was added. Finally, 59 items were composed and grouped into 12 domains. We asked the participants to rate the fit and necessity of each domain and the fit and importance of each item using the five-point Likert-type scale. The level of consensus was set to 80% of respondents indicating agreement [20].

### 2.7. Ethics

The study was approved by the Institutional Review Board of Yonsei University [YUWIRB-104189-202004-SB-040-03].

### 2.8. Data Analysis

The analysis of the first survey was performed by researchers in the occupational therapy department. In the first Delphi survey, the domain of self-management and its items to be surveyed were classified and organized.

The analysis of the second and third surveys were based on the values from the 5-point Likert scale. Content validity ratios (CVRs), averages, median, standard deviations, stability, reliability (Cronbach’s alpha), group stability (Mann–Whitney U test), and agreement were found using Microsoft Excel and SPSS 25 (IBM, Armonk, NY, USA).

In each survey, the minimum CVR was determined by the number of experts who participated [21]. According to the criteria, the CVR values of all items were defined as 0.31 for 31 panels in the second and third surveys. Stability, which is the panel’s agreement on each item, was analyzed by the coefficient of variation, which was divided by the arithmetic mean of each item’s standard deviation. If the coefficient of variation was less than 0.5, no additional Delphi survey was required, and a coefficient of variation of 0.5–0.8 indicated stability [21]. Additionally, if the coefficient of variation was 0.8 or higher, the results were considered erroneous and ignored [22].

## 3. Results

### 3.1. Demographics of the Panel Experts

The demographic characteristics of the final sample (*N* = 31) are presented in Table 1. Sixteen participants (52%) were female. Seventeen participants (55%) were in their 30s. Sixteen participants (51%) had six to eleven years’ work experience. Ten participants (32%) had three to five years of career experience in education in elderly care or a major in occupational therapy. Eight participants (26%) had three to five years’ experience in research about elderly or occupational therapy. The panel consisted of experts in occupational therapy and professors with a major in occupational therapy. In this Delphi survey, the group stability was demonstrated by showing *p* > 0.05 in all questions in the results of the Mann–Whitney U test.

### 3.2. Results of the First Delphi Survey

The stage 1 results are described in Table 2. Thirty-one participants accessed the first-phase survey and answered all questions. The original 12 domains were selected, and “Leisure activity management” was deemed a domain to be added. Thus, stage 1 results included 68 items in 13 domains.

### 3.3. Results of the Second Delphi Survey

In all domains, the CVR score was higher than 0.31, except for the “Paid or unpaid work” domain, which was deleted after discussion between researchers. The fit of two items, “Identifying the right medicine for you” and “Writing an application”, was below the minimum CVR value of 0.31, so they were also deleted after discussion among researchers. The importance of five items (“Managing sleep hygiene”, “Grading physical activity”, “Using an app related to transportation”, “Volunteering”, and “Writing an application”) had a CVR value of less than 0.31, so the “Paid or unpaid work” domain, its items, and four additional items were excluded from the third survey.

### 3.4. Results of the Third Delphi Survey

None of the 12 domains’ items analyzed had a minimum CVR of 0.31 or less, except the fit of three items and the fit and importance of two items (Table 3). After discussions between researchers, the five items were deleted. Finally, 12 domains and 54 items were derived. The IQR value of each item’s fit and importance is 0–2. The overall reliability value of the Delphi survey used in this study is Cronbach’s α = 0.984.

## 4. Discussion

In this Delphi study, specific occupation-based areas required for self-management were derived from occupational therapy studies, and the appropriateness and importance of sub-contents of each area were derived to systematically include the contents. The 12 occupation-based self-management areas used in the first Delphi survey were composed based on the lifestyle redesign intervention module for self-management of patients with chronic pain and its functions, developed by Simon and Collins [15]. The current study can provide a breakdown of the basis for occupational therapy intervention for chronically ill patients.

In the first Delphi survey, experts came up with opinions on the importance of managing the leisure activities of the elderly, which were consistent with the findings of a previous study [22] that leisure activity programs have an important effect on the elderly’s quality of life and participation in activities.

According to the results of the second Delphi survey, the content validity of “paid and unpaid work” was low, so it was excluded in the next survey. The low content validity might be due to cultural differences; it is not common for Koreans over 65 years to obtain new jobs [23]. The 12 occupation-based domains derived through the third Delphi survey are necessary domains for health promotion of the elderly; the domains of self-management of the elderly have similar contexts to derived balance, strength, sleep, nutrition, physical activity, eating habits, activity participation, etc. [24]. These results are significant in that the derived occupational therapy areas and activities are suitable for older patients with chronic diseases and thus selected as the necessary occupation-based care areas.

The elderly population is growing, and as such, legal and financial aid is needed to implement Korea’s “community-care” policies. However, there are no appropriate legal or payment systems for community-centered rehabilitation in Korea; these have only begun to be implemented in a few institutions, such as welfare centers for the disabled and public health centers [25,26]. Therefore, this Delphi study is of great significance as a preliminary study toward developing an intervention program for older patients with chronic diseases living in a community. As the main targets of occupation-based community-centered rehabilitation are people with physical disabilities who cannot be rehabilitated or people with brain lesions [27], the interventions focusing on health management for chronically ill patients are insufficient. Therefore, based on the results of this study and in conjunction with theories related to self-management, it is necessary to develop a concrete and systematic program about self-management for the elderly in the future.

This study proposed areas of self-management for elderly people. The main strengths of our study are the responses from professionals in occupational therapy and the fact that the response rates were very good. In addition, the group stability was derived by making the subjects of the second Delphi and third Delphi the same, and the reliability of the Delphi survey used in this study was proved to be more than 0.7 in Cronbach’s α.

However, this study has a few limitations. First, we analyzed the content validity of self-management items only with experts in occupational therapy. Therefore, reliability research should be conducted in the future. Second, only experts from South Korea were invited to participate, so our panel of experts was not international. Hence, this research represents a limited viewpoint.

## 5. Conclusions

This study used the Delphi method to develop the contents of a self-management program for elderly people with chronic diseases. The results include a total of 54 items divided into 12 domains. These results can help explain self-management by the elderly and clarify the contents that should be used in program development. Further research to confirm the reliability and development of a program using the results of this study are needed.

## Figures and Tables

**Table 1:** Demographic characteristics of the participants (*N* = 31).

Characteristics		Stage 1 *n* (%)	Stage 2 *n* (%)	Stage 3 *n* (%)
Sample size		31	30	30
Response rate		100%	100%	100%
Gender	Male	15 (48%)	15 (50%)	15 (50%)
	Female	16 (52%)	15 (50%)	15 (50%)
Age	20s	8 (26%)	8 (27%)	8 (27%)
	30s	17 (55%)	16 (53%)	16 (53%)
	40s	6 (19%)	6 (20%)	6 (20%)
Work experience	3–5 years	16 (51%)	15 (50%)	15 (50%)
	6–10 years	11 (35%)	11 (37%)	11 (37%)
	11 ≤ years	2 (6%)	2 (7%)	2 (7%)
Career in education	3–5 years	10 (32%)	9 (30%)	9 (30%)
	6–10 years	2 (6%)	2 (7%)	2 (7%)
Research experience	3–5 years	8 (26%)	8 (27%)	8 (27%)
	6–10 years	2 (6%)	1 (3%)	1 (3%)
Occupation (clinical/research/both)	Occupational therapist	19 (61%)	19 (63%)	19 (63%)
	Professor	12 (39%)	11 (37%)	11 (37%)

**Table 2:** Domain and items from the first Delphi survey.

Domain	Items (*N*)
Eating routines	5
Sleep management	5
Physical activity	8
Stress and mood management	5
Medication management	6
Energy and fatigue management	5
Time management	6
ADL and IADL management	6
Body mechanics and posture	5
Community integration	5
Social and relationship management	5
Paid or unpaid work	5
Leisure activity management	2
Total (*N*)	
13	68

**Table 3:** Fit and importance of self-management items for elderly people with chronic diseases (third Delphi survey).

Domain	Items	Fit				Importance			
		Mean	SD	CVR	Median	Mean	SD	CVR	Median
Eating routines	Managing regular meal times and eating habits	4.72	0.45	0.93	5.00	4.79	0.41	0.93	5.00
	Adjusting the amount of food	4.21	0.82	0.60	4.00	4.34	0.77	0.73	4.00
	Getting the right nutrients	3.93	0.70	0.40	4.00	4.17	0.85	0.53	4.00
	Understanding eating habits	4.34	0.72	0.67	4.00	4.55	0.74	0.67	5.00
	Controlling foods to avoid	4.38	0.56	0.87	4.00	4.59	0.68	0.87	5.00
Cronbach’s α		0.770				0.795			
Sleep management	Adhering to regular sleeping hours	4.62	0.56	0.87	5.00	4.62	0.62	0.80	5.00
	Maintaining a sleeping environment	4.34	0.61	0.80	4.00	4.48	0.69	0.73	5.00
	Managing sleep quality	4.28	0.70	0.67	4.00	4.41	0.73	0.67	5.00
	Knowing what to do before/after sleep	4.31	0.81	0.67	4.00	4.21	0.68	0.67	4.00
Cronbach’s α		0.792				0.792			
Physical activity	Exercising regularly	4.93	0.26	0.93	5.00	4.97	0.19	0.93	5.00
	Maintaining flexibility, strength, and endurance	4.59	0.57	0.87	5.00	4.66	0.55	0.87	5.00
	Stretching	4.62	0.49	0.93	5.00	4.72	0.53	0.87	5.00
	Doing exercises that suit you	4.90	0.31	0.93	5.00	4.93	0.26	0.93	5.00
	Identifying favorite physical activities	4.69	0.47	0.93	5.00	4.72	0.45	0.93	5.00
	Exercising in a safe environment	4.38	0.68	0.73	4.00	4.62	0.62	0.80	5.00
	Identifying sustainable exercise habits	4.62	0.56	0.87	5.00	4.72	0.53	0.87	5.00
Cronbach’s α		0.814				0.838			
Stress and mood management	Identifying the causes of stress	4.24	0.69	0.67	4.00	4.45	0.63	0.80	5.00
	Knowing stress-coping skills	4.62	0.62	0.80	5.00	4.62	0.56	0.87	5.00
	Evaluating the environment for inducing stress	3.93	0.88	0.13	4.00	4.10	0.82	0.40	4.00
	Checking mood	4.21	0.77	0.53	4.00	4.38	0.78	0.60	5.00
	Self-regulation and relaxation training	4.55	0.63	0.80	5.00	4.59	0.68	0.73	5.00
Cronbach’s α		0.879				0.904			
Medication management	Taking medication on time	4.93	0.26	0.93	5.00	4.90	0.41	0.87	5.00
	Checking the expiration date of the medicine	4.38	0.56	0.87	4.00	4.62	0.62	0.80	5.00
	Managing medication	4.76	0.44	0.93	5.00	4.79	0.49	0.87	5.00
	Education on drug abuse	4.24	0.64	0.73	4.00	4.55	0.63	0.80	5.00
	Using assistive tools related to taking medicine	4.48	0.69	0.73	5.00	4.52	0.69	0.73	5.00
Cronbach’s α		0.638				0.797			
Energy and fatigue management	Knowing your energy consumption	4.24	0.74	0.60	4.00	4.45	0.69	0.73	5.00
	Determining energy consumption	3.79	0.73	0.20	4.00	4.07	0.80	0.40	4.00
	Controlling energy and fatigue	4.79	0.41	0.93	5.00	4.76	0.44	0.93	5.00
	Using assistive tools to manage energy	4.52	0.69	0.73	5.00	4.48	0.69	0.73	5.00
	Knowing the proper alignment for energy conservation	4.55	0.74	0.67	5.00	4.55	0.69	0.73	5.00
Cronbach’s α		0.822				0.842			
Time management	Prioritizing	4.76	0.51	0.87	5.00	4.83	0.47	0.87	5.00
	Using assistive tools for schedule management	4.52	0.69	0.73	5.00	4.45	0.74	0.67	5.00
	Providing information about time balance	4.21	0.77	0.53	4.00	4.10	0.77	0.47	4.00
	Knowing the time to invest	3.97	0.87	0.20	4.00	3.86	0.83	0.13	4.00
	Balancing lifestyle	4.55	0.69	0.73	5.00	4.59	0.63	0.80	5.00
	Establishing future plans	4.52	0.63	0.80	5.00	4.52	0.63	0.80	5.00
Cronbach’s α		0.859				0.875			
ADL and IADL management	Knowing what you can do	4.72	0.65	0.87	5.00	4.79	0.49	0.87	5.00
	Figuring out what you can’t do	4.59	0.78	0.73	5.00	4.76	0.58	0.80	5.00
	Knowing how to use assistive tools	4.76	0.44	0.93	5.00	4.76	0.44	0.93	5.00
	Understanding the degree of help	4.69	0.60	0.80	5.00	4.69	0.54	0.87	5.00
	Utilizing community resources	4.72	0.59	0.80	5.00	4.69	0.60	0.80	5.00
	Using a compensatory strategy	4.76	0.51	0.87	5.00	4.72	0.53	0.87	5.00
Cronbach’s α		0.826				0.812			
Body mechanics and posture	Knowing the right posture	4.52	0.74	0.67	5.00	4.62	0.56	0.87	5.00
	Maintaining the right posture	4.62	0.68	0.73	5.00	4.66	0.48	0.93	5.00
	Identifying and maintaining one’s health status	4.59	0.63	0.80	5.00	4.66	0.61	0.80	5.00
	Managing pain	4.72	0.59	0.80	5.00	4.79	0.49	0.87	5.00
	Understanding body mechanics	4.17	0.85	0.53	4.00	4.17	0.85	0.53	4.00
Cronbach’s α		0.762				0.787			
Community integration	Knowing how to use public transportation	4.69	0.60	0.80	5.00	4.72	0.53	0.87	5.00
	Using a car (for those who can drive)	4.41	0.68	0.73	5.00	4.41	0.63	0.80	4.00
	Knowing the time required to a destination	3.86	0.74	0.27	4.00	3.97	0.82	0.27	4.00
	Knowing how to get to a destination	4.52	0.57	0.87	5.00	4.64	0.56	0.80	5.00
Cronbach’s α		0.797				0.760			
Social and relationship management	Maintaining your role	4.72	0.45	0.93	5.00	4.76	0.44	0.93	5.00
	Knowing how to deal with loss of relationships	3.86	0.79	0.20	4.00	4.24	0.74	0.60	4.00
	Using mobile phones and internet SNS	4.38	0.78	0.60	5.00	4.31	0.76	0.60	4.00
	Identifying areas of social activity	4.41	0.63	0.80	4.00	4.34	0.77	0.60	5.00
	Finding and participating in community activities	4.62	0.56	0.87	5.00	4.66	0.55	0.87	5.00
Cronbach’s α		0.819				0.834			
Leisure activity management	Identifying leisure activities	4.68	0.48	0.87	5.00	4.71	0.46	0.87	5.00
	Finding out about participating in leisure activities	4.79	0.42	0.87	5.00	4.75	0.44	0.87	5.00
Cronbach’s α		0.859				0.954			
Total Cronbach’s α		0.984							

## Data Availability

The data that support the findings of this study are available on request from the corresponding author, [H.Y.P.]. The data are not publicly available due to restrictions.
